# Phthalates in the diet of Mexican children of school age. Risk analysis

**DOI:** 10.1016/j.toxrep.2020.10.020

**Published:** 2020-10-29

**Authors:** María Magdalena García-Fabila, Araceli Amaya Chávez, Juan Carlos Sánchez Meza, Lilia Patricia Bustamante Montes, Alicia Reyes García

**Affiliations:** a Facultad de Química, Universidad Autónoma del Estado de México, Paseo Colón and Paseo Tollocan SN., Colonia Ocho Cedros, Toluca, Estado de México, C.P. 50120, Mexico; b Decanato de Ciencias de la Salud, Universidad Autónoma de Guadalajara, Av. Montevideo esquina Avenida Acueducto, Guadalajara, Jalisco, CP 44670, Mexico

**Keywords:** Food exposure, Diethyl phthalate, Dibutyl phthalate, Di-2 ethyl hexyl-phthalate, Children's Health, hazard index

## Abstract

Phthalates are widely used as plasticizers, additives, or solvents. Its extensive use has generated environmental and food contamination, which implies continuous population exposure. The aim of this work was to determine the probability of health risk of Mexican children exposed to phthalates through the consumption of contaminated food. A survey was applied to 384 Mexican school-age children (between 6 and 12 years old), to find out the type of food they eat most frequently, based on this, a research was made to know the concentration of phthalates contained in these foods. The daily intake had been calculated with the concentration of phthalates reported in food, obtaining: DEHP (19.50 μg/kg body weight/day), DnBP (5.52 μg/kg body weight/day) y for DEP (1.12 μg/kg body weight/day). The hazard index (HI) for DEP y DEHP was 0.49 to 42.5 for internal organs damage reported. HI for reproductive health damage due to exposure to DnBT and DEHP was of 0.04 to 5.58, so that there is a high probability that children's health is at risk. Therefore, it is necessary to a quantitative analysis of phthalates in food consumed in Latin American countries and establish the TDI of phthalates especially, to DEHP, which was obtained the higher HI.

## 1 Introduction

“Phthalates” are synthetic compounds derived from phthalic acid [1] that consist of an aromatic dicarboxylic acid where carboxy groups are found in adjacent carbons on a benzene ring [2]. These compounds have been used for more than 80 years [3], as industrial plasticizers, in the production of toys, bottles and pacifiers [2,4], as additives or solvents in shampoos, perfumes, nail polishes, cleaning products, paints, adhesives and pharmaceutical formulations [[4], [5], [6], [7]]. Also, they have been incorporated into the manufacturing process of medical devices and food packaging [[8], [9], [10]]. The use of containers to keep and pack food has been the primary source of phthalate migration [[11], [12], [13]]. Had been reported the presence of phthalates in food matrices such as dairy and meat in concentrations of 2.7 to 3350 μg/kg of fat [14], that when consumed by man constitute an important essential source of chronic exposure. There are 18 dialkyl phthalates compounds reported as plasticizers [15,16], the most frequent in foods are dimethyl phthalate (DMP), diethyl phthalate (DEP), di-isobutyl phthalate (DiBP), dibutyl phthalate (DBP), butyl benzyl phthalate (BBzP), diethyl hexyl phthalate (DEHP), dicyclo hexyl phthalate (DCHP) and di n-octyl phthalate (DnOP). The DEHP has been the one with the highest reported concentrations in all food groups (from 718 to 11,100 /g / Kg) [[11], [12], [13], [14],[17], [18], [19],[20], [21], [22], [23]].

Humans are exposed throughout life to different concentrations of phthalates, by dermal route using products that contain them as packaging and toys; by intravenously route using blood or intravenous tube fluids for packaged renal dialysis, by airway route in the workplace or indoor air where DEHP is released; by intake route when consuming or using well water near waste sites or by the consumption of food packaged in plastic, (fatty foods, dairy, fish, shellfish and, oils). Food phthalates exposure is one of the most important in children an intake of 4.68 μg/kg/day was reported and in adults 1.03 μg/kg/day [24].

After entering the body, the phthalic diesters are hydrolyzed to mono ester (more bioactive form) by esterase or lipases present in the salivary gland, intestine and, liver [25]. The aliphatic chains of phthalates undergo hydroxylation and oxidation reactions (type s-, s^-1^ and/or oxidation). In phase two of metabolism are formed conjugated with hydrophilic glucuronide, with the participation of the enzyme UDP-glucuronosyl transferase [25,26]. The elimination of compounds is through biological fluids mainly by urine, feces and, sweat, they have also been detected in amniotic fluid, placental tissue, hair, nails, semen and breast milk [[27], [28], [29], [30]].

[22] reviewed of works related to the content of phthalates in food, finding higher concentrations of DEHP (≥300 μg/kg) in poultry meat and dairy products up to 192.2 μg/kg (ice cream, cream and, cheese). With these values, the authors estimated the exposure of infants in the United States, based on real diets, finding that the intake dose exceeded the reference level in the USA [22].

Studies in different countries mention the relationship between food intake and phthalate exposure. In children between 6 and 11 years old, an association has been reported between the consumption of school lunch and the concentration of urinary phthalates of American children [31]. On the other hand, in a study in Taiwan, they found urinary concentrations of six different phthalates (DEP, DiBP, DnBP, BBzP, DiNP and DEHP), in boys aged 7 to 12 years old with mediums of 4.79 μg/kg bw/d and in girls of 2.62 μg/kg bw/d, attributing exposure to food intake [23]. The cumulative hazard index was greater than one, calculated with the reference dose for reproductive and hepatic damage [23]. In Belgium, the calculation of the daily intake (DI) of five phthalates (DEP, DnBP, DiBP, BBzP, DEHP) from quantification of urinary metabolites, reported values higher than the detection limit < LOD and 59.65 μg/kg bw/d, observing higher values for children than for adults [32]. This study suggests that for DEHP food intake is the main route of exposure. In 2019 a review of biomonitoring of phthalates was reported [33]. They reviewed studies in different countries, finding that the most dominant in the urine of children were through their primary metabolites mono methyl phthalate (MEP), Mono butyl phthalate (MBP), and DEHP, the latter was the compound with the highest concentration. This study performed an estimated daily intake (EDI) through the levels excreted in the urine, mentioning that the EDI max for DEHP and DBP was 8 and 0.8 μg/kg bw/d for the population of Taiwan, where the tolerable daily intake (TDI) values were exceeded by one or two orders of magnitude [33].

There are no studies that report the hazard index derived from the exposure of phthalates in food in México nor Latin America, so this work has the purpose of calculating the hazard index for phthalate exposure, taking as reference the reported content of these compounds in food, ingested by a Mexican child population.

## 2 Material and methods

### 2.1 Concentrations of phthalates in food

To establish the concentrations of phthalates in different foods, studies of dialkyl phthalates in food, development of analytical methodologies, and epidemiological studies that mentioned the presence of these compounds worldwide were reviewed. The research began with a bibliographic review of the development of analytical methodologies and epidemiological studies, which mention the presence of dialkyl phthalate. To search around the world, the Scopus, Mendeley, and Google Scholar platforms were used, the keywords were "Phthalates", "phthalates + foods", "Phthalates in Food", "Exposure to Phthalates", in journals published between 2001 and August 2019. The search served to establish the concentrations of phthalates in different foods. Documents that used specific analytical techniques with analytical quality controls were included. In addition, documents that reported foods not found in that study and subsequent articles were included. Some 34 documents were reviewed, although 14 of them were used for the final paper.

### 2.2 Mexican children's diet

**Fig. 1:**
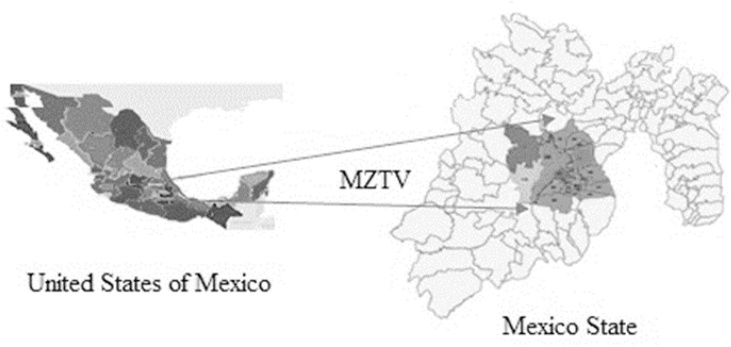
Metropolitan Zone of the Toluca Valley, (MZTV).

To knowledge the children's diet in central Mexico, this work studied the Metropolitan Zone of the Toluca Valley (MZTV), State of Mexico, second national place of population density, with coordinates 19 ° 21'15 "N99 ° 37¨51" OR. The ZMVT is composed of 15 municipalities (Fig. 1), with 2,116,506 inhabitants, of which 284,504 are children between 6 and 12 years old [34].

School-age children (6 to 12 years old) were chosen as the study population; this age, the community begins to make decisions about what they eat at home and school, and they have the possibility of make small purchases mainly of packaged and / or processed foods. The healthy eating habits are formed and observing them properly leads to a reduction in the risk of overweight, obesity and of non-communicable diseases in later stages of life [35].

A questionnaire [annex one] was elaborated, composed of three sections: 1) an identification form, and general data, 2)a 24 -h consumption section and 3) a food consumption frequency section based on the essential Mexican diet [36]. The equation 1, describes the sample size calculation A questionnaire [annex one] was elaborated, composed of three sections: 1) an identification form, and general data, 2)a 24- hour consumption section and 3) a food consumption frequency section based on the essential Mexican diet [36]. Equation 1 describes the sample size calculations.

(1) $$n=\frac{NZ^{2}pq}{d^{2}\left(N-1\right)+Z^{2}pq}$$

Where: N = population size (284,503.8); Z = 95% critical Z value (1.96). d = absolute precision level (0.05). p = proportion of the population that will accept answering the questionnaire (0.5); q = proportion of the population that will not accept answering the questionnaire (0.5) [37,38], obtaining a sample size of n = 384.

The questionnaire was applied to children between 6 and 12 years old they attend primary schools located in the five municipalities with the highest population of the ZMVT (Almoloya de Juárez, Metepec, Lerma, Toluca, and Zinacantepec), previous authorization of the dean schools. The children answered the questionnaire with their parents.

### 2.3 Dose of exposure by ingestion

To calculate the dose of phthalate exposure (DEP, DBP and DEHP) per intake (EDI in mg/kg bw-day); Equation 2 was used for each food.

(2) $${EDI}_{food=}\frac{CxTIxEF}{bw}$$

Where: C = phthalate concentration in the food, TI = Intake rate, bw = body weight (30.1 kg, average weight obtained from the questionnaires) and exposure factor (EF) that was calculated using equation 3.

(3) $$EF=\frac{\text{e}\text{x}\text{p}\text{o}\text{s}\text{u}\text{r}\text{e}\;\text{f}\text{r}\text{e}\text{q}\text{u}\text{e}\text{n}\text{c}\text{y}\;x\;\text{e}\text{x}\text{p}\text{o}\text{s}\text{u}\text{r}\text{e}\;\text{d}\text{u}\text{r}\text{a}\text{t}\text{i}\text{o}\text{n}}{\text{e}\text{x}\text{p}\text{o}\text{s}\text{u}\text{r}\text{e}\;\text{t}\text{i}\text{m}\text{e}}$$

### 2.4 Calculation of Hazard quotient (HQ) and Hazard index (HI)

To determine the probability of an adverse effect in the study population, the exposure dose of each phthalate was estimated by food consumption (EDI_oral_). With these data, the hazard quotient (HQ_oral_) was calculated for a critical non-cancer effect using equation 4.

(4) $${HQ}_{oral}=\frac{{EDI}_{oral}}{{RfD}_{oral}}$$

The oral reference dose (RfD_oral_) according to the EPA, is an estimate of the daily intake of a xenobiotic by the human population (including sensitive subgroups) throughout their lives without having a considerable risk of harmful effects, this value is usually derived from a No Observed Adverse Effect Level (NOAEL), Lowest Observed Adverse Effect Level (LOAEL) or the lowest value of the 95% confidence interval of the lower dose at which a measurable adverse effect is observed in the dose response (BMDL) relationship [[39], [40], [41]] with uncertainty factors that reflect the limitations of the research data where they come from [42]. Oral Reference doses (RfD in mg/kgbw-day) were obtained from the Agency for Toxic Substances and Disease Registry (ATSDR). For DEHP, RfD is 0.02 mg/kg/day based on a LOAEL of 19 mg/kg/day for chronic oral intake based on a hepatic effect of guinea pigs fed on a diet containing DEHP for one year [43]. On the other hand, [44] calculated an RfD of 0.3 mg/kg-day for DEHP related to the decrease or absence of male reproductive organs in Sprague-Dawley rats with a BMDL10 of 27 mg/kg-day.

For the DBP, the RfD of 0.1 mg/kg/day obtained from a NOAEL of 125 mg/kg-day and LOAEL of 600 mg/kg-day was considered due to increased mortality in rats, incorporating an uncertainty factor of 1,000. [45] and RfD of 0.3 mg/kg-day related to decreased fetal testosterone in Sprague-Dawley rats pregnant with a NOAEL of 30 mg/kg-day and a LOAEL of 50 mg/kg/day [46].

For the DEP, the RfD of 0.8 mg/kg/day based on the decrease of the growth rate and the alteration of the organ weight in rats with an uncertainty factor of 1,000 [47].

The hazard index (HI) was obtained with the sum of all hazard quotients for a specific critical effect, using equation 5. Values greater than one mean that there is a probability of presenting the harmful effect from the consumption of food contaminated with phthalates in the exposed population [44].

(5) $$HI={HQ}_{oral\;DEP}+{HQ}_{oral\;DBP}+{HQ}_{oral\;DEHP}$$

## 3 Results

### 3.1 Concentration phthalates in food

Reports of contamination foods were found with dialkyl phthalates (DEP, DBP and DEHP) and were grouped into 8 categories that correspond to the basic Mexican basic diet [36,48]: 1. Groceries, 2. Oils and fats, 3. Dressings and sauces, 4. Poultry and meat, 5. Cereals and tubers, 6. Fruits, vegetables and vegetables, 7. Fish and seafood and 8. Sausage and dairy products.

Fifteen studies that were found in the search period including [21] and [22]. These authors include more than 35 studies that report concentrations of phthalates in foods typically consumed by the general public, used their information of phthalates, and some of the original sources were reviewed. The concentrations of DEP, DBP and DEHP were identified for each food.

DEHP was the compound that presented the highest concentration, in all the cases reported, the foods with the highest content were: ham, oils, and fats followed by milk cream, condiments, cheese and poultry, with average concentrations per food of 710 a 1378.2 μg/kg, Table 1.

**Table 1:** Average concentrations and intervals of DEP, DBP and DEHP in μg/kg, by food category.

Category	Food	Studies	DEP _average_	min	max	DBP _average_	min	max	DEHP _average_	min	max
A Groceries	Bread	10	*5.2*	0	50	41.9	0	5130	318	0	1960
	Eggs	4	1.9	0	11.8	1.6	0.6	100	101	0	600
	Juice	3	0	0	0	10.75	0	560	69.8	0	1700
	Drinks alcohol free	5	18	0	90	1.23	0	190	15.4	0	110
	Bottled water	8	0.01	0	0.4	0.47	0	2	4.22	0	30
AG Oils and fats	oils, butter and fats	11	14.8	0	180	603.7	0	10600	1299	0	11900
AS Dressings and sauces	nuts and spreads	3	5.02	0	23.3	82.1	43	17460	307.3	143	19860
	seasonings	4	42.39	0.11	84	47.65	0.81	155	748.2	5	2154
B Meat	Chicken	7	3.68	0	67.3	23	0	200	710	2.6	720
	Beef	8	1.6	0	8.9	260.8	0	6200	198.4	0	2500
	Pork	9	1.21	0	25.7	7.14	0	300	279.4	0	3700
C Cereals and tubers	Cereals and Cereal Products	12	42	0	2200	150.4	0	2100	281	0	4420
	Pasta, Noodles and Rice	7	1.2	0	22.4	21.1	0	360	74.9	0	761.6
D Fruits and Vegetables	Vegetables and Vegetables Products	11	2.31	0	9	10.82	0	630	94.7	0	2200
	Fruits and fruit products	10	10.2	0	730	18.7	0	120	87.5	0	1200
E Fish and seafood	Seafood	9	3.2	0	340.3	24.98	0	500	250.2	0	5933
F Delicatessen and dairy products	Yogurt	8	0.45	0	1.9	1.7	0	5.8	47.16	0	192.2
	Ice cream	6	0.35	0	1.9	0.72	0	4.8	309.4	10.2	390
	Cheese	8	12.4	0	271.9	105.5	0	513	742.5	0	550
	Milk	11	1.75	0	15.5	15.36	0	225.8	43.19	0	108.6
	Cream	6	1.57	0	5.6	240.7	0	350.6	784.28	180	1400
	Ham	3	0.59	0.2	0.55	73.6	0.23	147	1378.2	5.5	3740
	Sausage	3	0.55	0.2	0.59	0.97	0.7	1.5	121.03	3.7	300
	Chorizo	1	-	-	-	0.27	1.11	3.71	2.14	0.27	2.14

The DBP has average concentrations of 603.7 μg/kg in fats and oils, it should be mentioned that the intervals found are very wide, in the case of this type of food the maximum value was 17.6 times greater than the average value. DBP has average concentrations of 603.7 μg/kg in fats and oils; it is important to mention that the range found are very wide, in the case of this type of food the maximum value was 17.6 times greater than the average value.

It is worrying that processed cereals of high consumption by the children of the Mexican population have values up to 2100 μg/kg, in products made in countries such as the United States, Germany, China, Japan, or Canada.

Processed cereals produced in countries such as the United States, Germany, China, Japan, or Canada, have values ​​up to 2100 μg / kg, and children of the Mexican population, this is worrying. The DEP is the compound that was found with the lowest concentrations reported in food. The content ranges from 0 to 42.39 μg/kg, although the maximum concentrations reported were 2200 μg/kg in cereals and bakery products.

### 3.2 The diet of Mexican children

Table 2 integrate the results of the consumption of each food and its contribution of phthalates per day. The foods of greater consumption by the children of school age (from 6 to 12 years) of the MZTV, correspond to six categories from the eight categories selected and the phthalates contents were: groceries (1.72 kg/day), fruits, vegetables and vegetables (0.72 kg/day), cereals and tubers (0.65 kg/day), sausage and dairy products (0.576 kg/day), meat products (0.18 kg/day) and fats and oils (0.005 kg/day). The fried foods were considered in the calculation of the daily intake of fats and oils, due to their high oil content.

**Table 2:** Daily intake of phthalates per food.

Category	Food	Portion (kg /day)	Concentration (μg/kg)			Daily dose = TI x C (μg/day)		
			DEP _average_	DBP _average_	DEHP _average_	DEP	DBP	DEHP
A	Bread	0.23	5.2	41.9	318.0	1.2	9.6	72.7
	Egg	0.13	1.9	1.6	101.0	0.2	0.2	13.1
	Water	1.00	0.0	0.5	4.2	0.0	0.5	4.2
	Soda	0.25	18.0	1.2	15.4	4.5	0.3	3.9
AG	Cooking oil	0.005	14.8	603.7	1299.0	0.1	3.0	6.5
	Fried food	0.05	NR	14.1	111.6	0.0	0.8	6.1
B	Chicken	0.18	3.7	23.0	710.0	0.7	4.2	129.6
C	Pasta	0.20	1.2	21.1	74.9	0.2	4.2	15.0
	Cereals	0.46	42.0	150.4	281.0	19.1	68.4	127.9
D	Fruits	0.63	10.2	18.7	87.5	6.4	11.7	54.7
	Vegetables	0.10	2.3	10.8	94.7	0.2	1.1	9.5
F	Pasteurized milk	0.375	1.8	15.4	43.2	0.7	5.8	16.2
	Yogurt	0.200	0.5	1.7	47.2	0.1	0.3	9.4
	Milk cream	0.001	1.6	240.7	784.3	0.0	0.2	0.8

The foods of higher consumption by the children of school age (from 6 to 12 years) of the MZTV correspond to six categories from the eight categories selected, and the phthalates contents were: groceries (1.72 kg/day), fruits, vegetables and vegetables (0.72 kg/day), cereals and tubers (0.65 kg/day), sausage and dairy products (0.576 kg/day), meat products (0.18 kg/day) and fats and oils (0.005 kg/day). The fried foods were considered in the calculation of the daily intake of fats and oils, due to their high oil content. Table 2 integrates the results of the consumption of each food and its contribution to phthalates per day.

The hazard quotient (Table 3) was calculated considering that the food was ingested 365 days a year. The average body weight of a child between 6 and 12 years old, obtained from the surveys, was 31.3 kg and considering the reference doses reported by the EPA and ATSDR.

**Table 3:** Estimated Daily Intake (EDI μg/kg body weight/day), hazard quotient (HQ) and Risk Index (HI).

Category	EDI average			EDI maximum		
	DEP	DBP	DEHP	DEP	DBP	DEHP
A	0.19	0.34	2.99	1.14	39.34	18.59
AG	0.02	0.99	2.26	0.29	16.95	19.68
B	0.02	0.13	4.13	428.85	1543.87	1522.43
C	0.62	2.31	4.55	32.04	32.74	68.93
D	0.22	0.44	2.35	14.59	6.40	37.92
F	0.03	0.27	1.14	0.20	2.76	3.27
Total	1.1	4.21	17.42	477.11	164.06	1670.82
HQ_org_* HI_org_*	0.001	NC	0.87	0.6	NC	83.54
	0.871			84.14		
HQ_fer_** HI_fer_**	NC	0,01	0,06	NC	5.47	5.57
	0.07			11.04		

* HQ_org_ hazard quotient for alterations in internal organs.

** HQ_fer_ hazard quotient for fertility effects. NC = not calculated.

The average body weight of a child between 6 and 12 years old, obtained from the surveys, was 31.3 kg and considering the reference doses reported by the EPA and ATSDR. The hazard quotient was calculated considering that the food was ingested 365 days a year, Table 3.

**Fig. 2:**
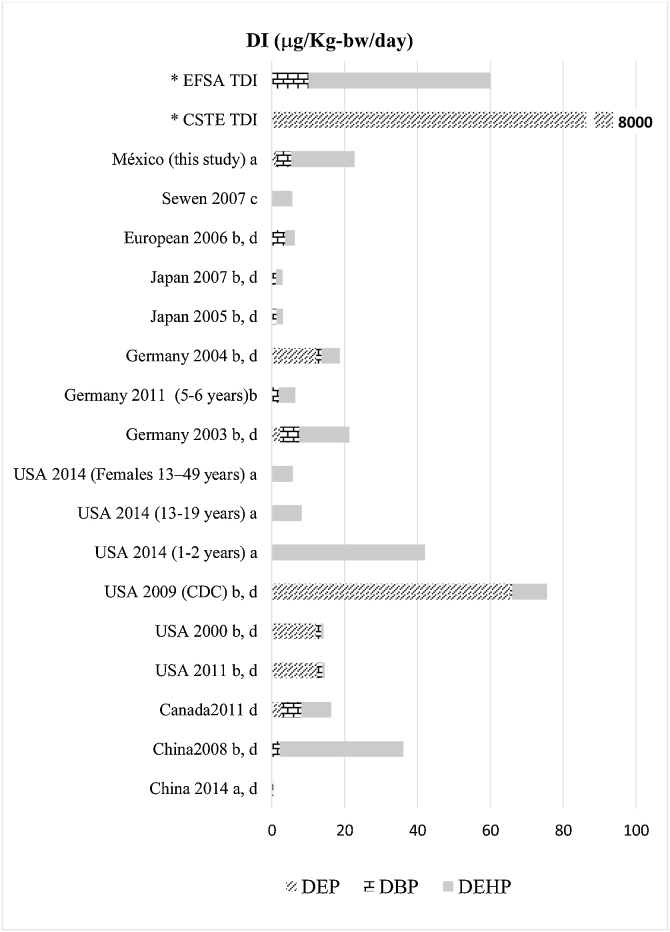
Daily intakes (DI) of DEP, DBP, and DEHP in different countries. a. Daily intake calculated from the concentrations in media; b. Daily intake calculated from urinary metabolite; c. Daily intake calculated by the ACC-humanmodel; d. Daily intake calculated for the general population; DEP. Dietyl phthalate; DBP. Dibutyl phthalate; DEHP. Di-2 etyl hexyl phthalate; EFSA. European Food Safety Authority; CSTE. EU Scientific Committee for Toxicity, Ecotoxicity and the Environment; TDI. Tolerable Daily Intake.

Source: ([[61], [62], [63], [64], [65], [66]], [15,49,52,67], [22,50,68,69]).

The estimated average daily intake in this study was higher for the DEHP 17.42 μg/kg bw/day, followed by the DBP 4.21 μg/kg bw/day and the DEP 1.1 μg/kg bw / day, results similar to those of [49] who calculated a daily intake of 13.8 μg/kg bw/day for DEHP, 5.2 μg/kg bw/day for DBP and 2.32 μg/kg bw/day for DEP in a general German population, their study calculated the intake using urinary metabolite excretion factors. In 2011 Koch reported a study where he analyzed a German community of children from 5 to 6 years old through a cumulative risk study, obtaining an intake dose of 4.5 μg/kg bw/day for DEHP and 1.9 μg/kg bw/day for the DBP, the DEP was not analyzed [50]. The Koch results showed a reduction of about 60% in DBP and DEHP exposure in the German population in 8 years. In a study in the USA [51], daily intake of DEHP was 9,472 μg/kg bw/day, 0.665 μg/kg bw/day for DBP and 65.4 μg/kg bw/day for DEP, this study shows the higher exposition of DEP, even so within the values ​​accepted by the CSTE. Serrano in 2014 calculated the DI for the DEHP using a similar methodology like this study using the reported concentrations of phthalates in food; they observed that the populations of children are exposed to concentrations of 5.1 to 7.4 times higher than those of adolescents or women of reproductive age respectively. Other studies related to DI phthalates from different countries will be compared in Fig. 2.

The exposure of each population is defined by the habits of local food consumption, although the food can come from anywhere of the world. The exposures reported for the United States of DEP are greater than those estimated for Mexico and the low exposure in 2014 in China is surprising, the reason can be that the country has regulations related to the use of phthalates in food packages. [52].

The European Food Safety Authority (EFSA) considers a tolerable daily intake (TDI) of 50 μg/kg bw/day for DEHP and 10 μg/kg bw/day for DBP [33]. The EU Scientific Committee for Toxicity, Ecotoxicity and the Environment (CSTEE) reports a TDI of 37 μg/kg bw/day for DEHP and 8000 μg/kg bw/day for DEP for the general population. Considering these data, the IDE in this study for DEHP, DBP, and DEP is within acceptable parameters taking account of the average calculated values. On the other hand, when the maximum values ​​reported in food by different studies were considered, higher daily intakes of phthalates are observed (Table 3), which implies a risk for the population that consumes them, especially in the case of DEHP. The maximum intake may be 45 times higher than the suggested concentration of DEHP by the CSTE or 33.4 times higher than indicated by the EFSA. In case of DBP if the maximum value is ingested, this may represent 16.4 times higher than the recommended concentration for this compound by the EFSA.

[29] reported a review of documents about the toxicity of phthalates, presenting 13 studies carried out between 2001 and 2014, observing that the trend between the values ​​of daily intake (DI) throughout this period is harmful for the DEHP, Katsakantami et al., suggest that strict regulations on the use of phthalates in various countries have promoted the decrease in DI.

The average hazard quotient HQ calculated for the DEHP, DBP, and DEP do not represent values ​​higher than the unit, so it can be considered that there is no risk of food intake in the MZTV (Table 3). However, the HQ calculated with the maximum phthalate values reported are above the unit for the DEHP and the DBP, except for the DEP.

The Hazard index was calculated for two potential effects, the first damage on internal organs HI_org_* = 0.871, obtaining a maximum value of 84.14 the intake of food contaminated with DEP and DEHP. The second for reproductive health effect with an HQ_fer_ = 0.07 with a maximum value of 11.04 calculated the intake of food contaminated with DBP and DEHP in Mexican children of school age. [44], mentions that a value less than the unit of HI means that it is unlikely that the adverse effects of exposure to chemicals will be manifested in the population. The HI values ​​between 1 and 100 indicated that there is a possibility that adverse effects will be observed. The average values ​​calculated for HI do not exceed the unit, but the maximum values ​​are between 1 to 100, indicating that there is a health risk for Mexican children.

## 4 Discussion

Foods with high fat content such as oil and obese t groups, and dressings and sauces are the ones with the highest concentrations of DEP, DBP, and DEHP, Table 1. The oils usually are extracted by extrusion of hot or cold vegetable seeds [53] and butter is a process in which the fattiest part of milk is getting [54]. These oily fractions could extract phthalates from containers or hoses during their production or storage process due to their lipophilicity (K_ow_ from 2.47 to 5.03)) [43,45,47] and other factors such as temperature and the contact time.

One of the foods with the highest infant consumption are cereals (Table 2). According to the surveys applied, Mexican children consume an average of 0.66 kg of cereals per day. In this type of food, concentrations between zero and 4420 μg / kg of DEHP were found; the highest concentration was reported in North America (Serrano. et al., 2014). Likewise, this group presented the highest levels of DEP between zero and 2200 μg/kg, concentration reported in Canada (Serrano. et al., 2014), so monitoring in production, transport, and storage of this food are necessary to prevent health risk to children.

DEHP is the compound that was reported in all the studies analyzed, in all food groups and with the highest concentrations, mainly in chicken > cereals > bread, Table 2, these foods are the most consumed by children. Exposure to DEHP is associated with gynecomastia in boys, premature onset of puberty in girls and attention deficit hyperactivity disorder [8,26,33,[55], [56], [57]].

In the present study, the HQ was calculated by taking the reference doses reported by the EPA for effects on internal organs produced by DEP and DEHP (HQ_org_) and the values ​​for fertility effects produced by DBP and DEHP (HQ_fer_), for estimation of HQ the RfD reported by [44] was used in this work. The calculated values ​​of HI represent a risk for oral exposures at high concentrations of DBP and DEHP in terms of reproductive health and effects on internal organs due to the intake of food contaminated with DEP and DEHP. The hazard index estimate has been evaluated by calculating the hazard quotient HQ for a specific effect, such as the androgenic damage reported by [23] for a Taiwanese population. These authors observed HQ_fer_ with values ​​higher than one for children between 7 to 12 years for six phthalates, and subsequently, the HI of liver damage was calculated for BBzP, DiNP, and DEHP and reproduction for DBP, DiBP, DEHP and BBzP observing values ​​above one in all cases, this study considered a severe s exposure to DBP, DiBP and DEHP in Taiwan especially in young children. [32], estimated exposure with urinary metabolites in children from 1 to 12 years and adults from 13 to 83 years observing variations when calculating HQ used the TDI or the RDf. The differences in HI_TDI_ (0.55 to 2.80) were 3 to 4 times greater than with HI_RfD_ (0.16 to 0.74), so to make comparisons the calculation will have to be homologated, in general it is mentioned that the HI values ​​were higher in children than in adults In studies carried out in Europe are mentioned that between 15 and 30% of German or Danish children the hazard index was higher than one [50,58,59].

According to the present study it is necessary to perform quantitative analyzes of phthalates contents in the different categories of foods consumed by Latin American countries, and promote the regulation of limits of these compounds to avoid damage to children’s health, especially to DEHP, that it is present in all food groups with the highest concentrations.

Also, it is important to develop studies related to the practices of food handling. Food handling is done with plastic gloves to avoid microbial contamination; however, this also implies contamination of the final food as observed in China [52]. It has been reported that the use of phthalates has reduced since 2010, using alternative plasticizers in materials used for walls or ceilings, [60] reviewed the use of 20 alternative plasticizers to phthalates in plastic materials, some of them made to from vegetable oils, while others are synthetic esters, among their results.

Phthalates are compounds with extensive use, the lack of regulations allows them to be used in any process and can be incorporated into any product, whether associated with food or not, so the review and control of production methods, packaging materials and the consumption of unprocessed foods are points that should be considered for the prevention of health risks.

## 5 Conclusions

The consumption of food with high-fat content such as oils, butter, and fried foods increase the probability of phthalate intake, especially DEHP, DBP, and DEP.

Bread and cereals are a source of exposure to phthalates such as DBP and DEHP, given the high frequency of consumption and their reported phthalate content.

The estimated daily intake of phthalates, calculated for MZTV children between 6 and 12 years old, with the concentration of phthalates consulted in the bibliography was higher for DEHP (19.5031 μg/kg/day), followed by DBP (5.5197 μg/kg/day) and DEP (1.12 μg/kg/day).

The Hazard Index obtained for the critical effects of damage to internal organs are in the range of 0.49 to 42.52 for DEP and DEHP. For reproductive health effects between 0.04 and 5.58, for DBP and DEHP, which indicates that children who consume food contaminated with the maximum daily intakes calculated for this type of phthalates may be at risk.

Phthalates are compounds with extensive use, the lack of regulation allows them to be used in any process and can be incorporated into any product associated with food

The review and regulation of production methods, packaging materials, and consumption of unprocessed foods are points that should be considered for the prevention of health risks in Latin American countries.

## Author statement

Conception and design of study: A. Amaya Chávez, M.M. García Fabila, L.P. Bustamante Montes.

Acquisition of data: M.M. García Fabila, A. Amaya Chávez.

Analysis and/or interpretation of data: A. Amaya Chávez, M.M. García Fabila, L.P. Bustamante Montes. J. C. Sánchez Meza, A. Reyes García.

Drafting the manuscript: M.M. García Fabila, A. Amaya Chávez.

Revisiting the manuscript critically for important intellectual content: L.P. Bustamante Montes. J. C. Sánchez Meza, A. Reyes García.

Approval of the version of the manuscript to be published (the names of all authors must be listed): A. Amaya Chávez, M.M. García Fabila, L.P. Bustamante Montes. J. C. Sánchez Meza, A. Reyes García.

## Declaration of Competing Interest

The authors report no declarations of interest.
